# A field polymerizing hydrogel enables simultaneous antimicrobial, hemostatic, and analgesic delivery in traumatic wounds

**DOI:** 10.1038/s41598-026-37521-y

**Published:** 2026-02-02

**Authors:** Elizabeth A. Pumford, Christopher D. Hamad, Amaka I. Enueme, Zeinab Mamouei, Nicholas Peterson, Christopher Hart, Chad Ishmael, Alan Li, Rahul Sobti, Jack Pearce, Jeremiah Taylor, Micah Ralston, Jared D. Wainwright, Kan Nakamoto, Perenlei Enkhbaatar, Kaitlyn A. Cook, Kevin P. Francis, John Adams, Alexandra Stavrakis, Joseph C. Wenke, Andrea M. Kasko, Nicholas M. Bernthal

**Affiliations:** 1 Department of Bioengineering, University of California, Los Angeles, Los Angeles, CA 90095 USA; 2 Department of Orthopedic Surgery, David Geffen School of Medicine, University of California, Los Angeles, CA 90095 USA; 3 David Geffen School of Medicine, University of California, Los Angeles, Los California, CA 90095 USA; 4 University of California, Los Angeles, Los Angeles, CA 90095 USA; 5 Department of Orthopaedic Surgery and Rehabilitation, The University of Texas Medical Branch, Galveston, TX 77555 USA; 6 Department of Anesthesiology, The University of Texas Medical Branch, Galveston, TX 77555 USA; 7 Department of Orthopaedic Surgery and Rehabilitation, Shriners Children’s Texas, the University of Texas Medical Branch, Galveston, TX 77555 USA; 8 Department of Orthopaedic Surgery, University of California, Los Angeles, 1225 15th Street-Suite 3144B, Santa Monica, CA 90404 USA

**Keywords:** Biotechnology, Drug discovery, Medical research, Microbiology

## Abstract

Traumatic injuries in resource-limited settings, such as remote, rural, or disaster-affected environments, require wound care solutions that can effectively address hemorrhage, infection, and pain outside of traditional clinical infrastructure. We developed a field-polymerizable hydrogel wound dressing capable of delivering tranexamic acid (hemostatic), vancomycin and tobramycin (broad-spectrum antibiotics), and lidocaine (analgesic) directly to the site of injury. Using computational modeling, we designed a lightweight, rugged hydrogel that polymerizes rapidly with potable water and conforms to irregular wound beds. The system demonstrated burst release of hemostatic and analgesic agents and sustained antibiotic release over four days. In vitro and in vivo testing confirmed the hydrogel’s ability to stabilize clots, prevent fibrinolysis, and eradicate polymicrobial gram-positive and gram-negative bacterial infections in a murine model of open fracture. Large animal studies further validated its translational potential in a large, complex wound. This modular, multifunctional platform provides a field-ready solution for wound management in austere environments, with the potential to reduce infection, control bleeding, and improve wound stabilization when access to definitive care is delayed.

**Supplementary Information:**

The online version contains supplementary material available at 10.1038/s41598-026-37521-y.

## Introduction

In urban, remote, austere, or disaster-affected environments, medical care is often delivered outside traditional clinical settings to temporize traumatic injuries and manage infection, hemorrhage, and pain. Open lower extremity fractures are common in these contexts and are associated with infection and non-union rates as high as 20 and 40%, respectively^1–11^. These complications can lead to limb loss or death, particularly in polytraumatized patients^8–10^.

Early systemic antibiotic administration—ideally within the first 1 to 3 h of injury—is the single most important factor in reducing infection risk^12–14^. However, there is growing evidence supporting the use of local antibiotic delivery as an effective adjunct in open fracture management^15^. Pathogens such as *Staphylococcus aureus*, *Escherichia coli*, and *Pseudomonas* spp. are common wound contaminants, originating from either the environment or the patient’s own microbiota^16^. These organisms may coexist synergistically, enhancing colonization and persistence, and increasing the risk of amputation and mortality^17–19^.

Hemorrhage accounts for 30–40% of trauma-related deaths within the first 6 h and is the leading cause of death in pelvic trauma^20–22^. In cases involving the pelvic venous plexus, blood loss can reach 1 L per minute if not promptly controlled. Long bone injuries, including femoral and tibial shaft fractures, can result in 1–1.5 L of blood loss. Field management typically relies on pressure dressings, tourniquets, or commercially available hemostatic dressings such as QuikClot™.^23,24^ However, tourniquet use carries risks, including ischemia-reperfusion injury and compartment syndrome, which can complicate limb salvage efforts.

Despite the pressing need, field wound care has seen limited innovation in recent decades due to the challenge of addressing multiple clinical priorities under real-world constraints. Ideal dressings for austere environments must be lightweight, rapidly forming, conformable, and rugged. Hydrogels, especially those derived from polyethylene glycol (PEG), offer promise as both wound dressings and drug delivery platforms. Their high-water content, biocompatibility, and tunable mechanical properties allow for controlled, sustained therapeutic release by modifying mesh size and crosslink density^25,26^. There has been recent growth in the field of advanced and stimuli-responsive hydrogel wound dressings, though these systems often fall short when it comes to affordability, stability in extreme environments, and ability to gel in situ^27,28^.

To address the multifactorial challenges of infection, hemorrhage, and pain, we developed a field-polymerizable PEG hydrogel capable of simultaneously delivering vancomycin, tobramycin, tranexamic acid (TXA), and lidocaine^29–31^. The hydrogel is activated using only potable water, without the need for specialized equipment or light-based polymerization, making it uniquely suited for field application. The system was engineered to meet key constraints: minimal weight, environmental stability, and broad-spectrum antimicrobial activity.

**Fig. 1:**
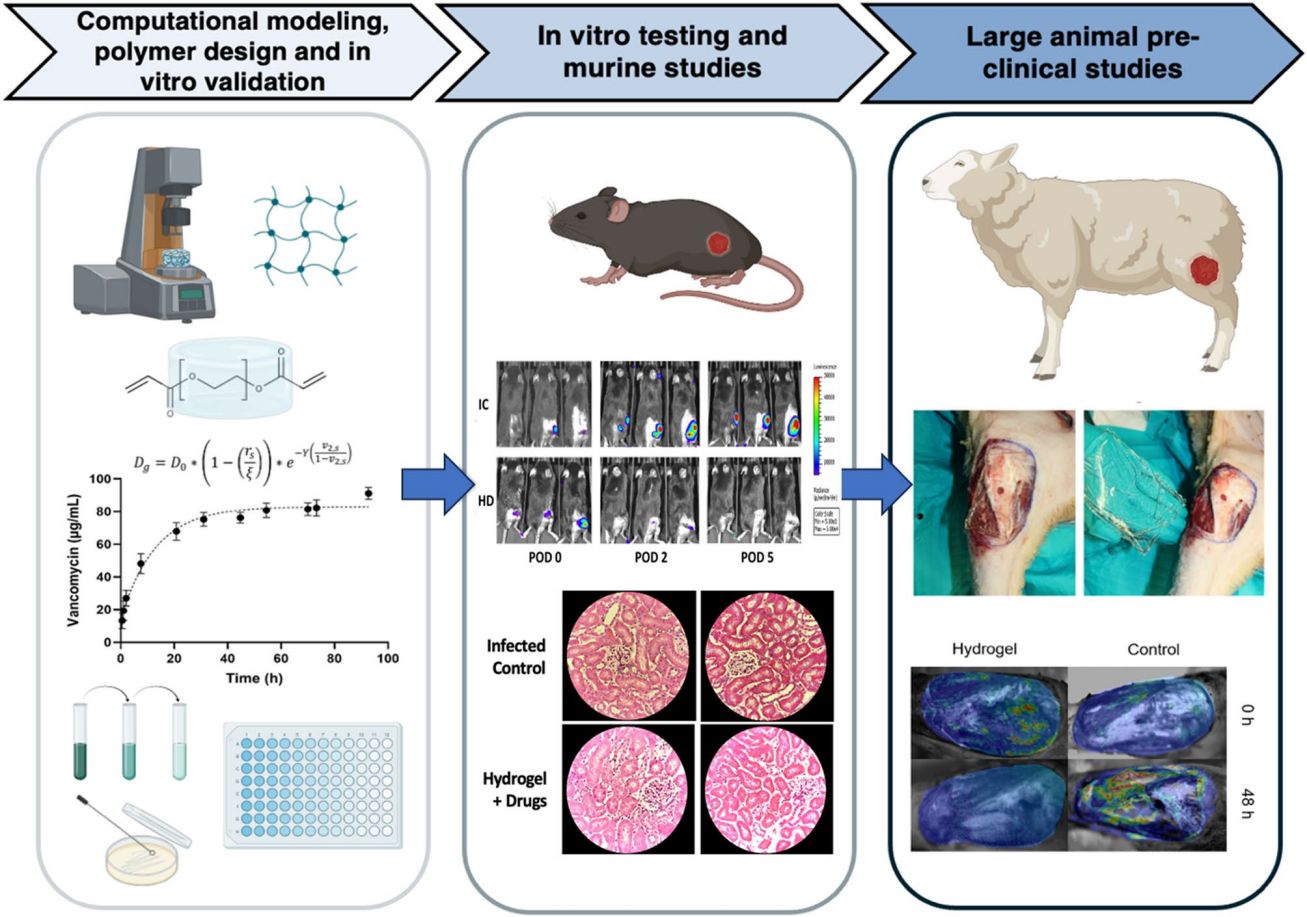
Research workflow diagram illustrating the sequential steps to develop and validate the hydrogel platform, highlighting the iterative feedback loops. The research progressed from initial computational modeling and in vitro validation, through to small animal in vivo testing, and culminating in large animal preclinical studies.

Our development workflow (Fig. 1) included computational modeling, materials characterization, and in vitro drug release assays, followed by efficacy testing in both small and large animal models. The platform leverages well-established PEG chemistry, using a starting material that is FDA-approved as a laxative, enabling predictable drug release and facilitating future clinical translation^32^. Localized drug delivery reduces the risk of systemic toxicity, resistance, and opioid dependence. Furthermore, the hydrogel is modular and can be readily adapted to incorporate alternative therapeutics (e.g., antifungals), providing a versatile platform for field medicine and trauma care in resource-limited settings^33–35^.

## Results

### Computational modeling—cumulative release simulations

Computational modeling of diffusion of therapeutic agents from hydrogels can aid in the design of hydrogel characteristics to achieve the desired release profiles (*Supplemental Methods*,* S1. Computational Modeling*). Diffusion of drugs from a hydrogel depends on the gel’s volumetric swelling ratio (Q_v_), polymer volume fraction (v_2,s_), and specific volume of polymer (*v*). After calculating the molecular weight between crosslinks (M_c_), the root mean square end-to-end distance of polymer chains between 2 neighboring crosslinks can be determined and the mesh size (ξ) can be calculated. These values were used with drug diffusion coefficients (D_0_), calculated using the Stokes-Einstein equation, to estimate the independent diffusion of each drug from the hydrogel mesh, (D_g_, *Equation S7*). Modeling hydrogels as thin films of height “*L*”, therapeutic drug concentration as a function of time was calculated according to Eq. 1.

1 $$\frac{{{M_t}}}{{{M_\infty }}}=1 - \sum\limits_{{n=0}}^{\infty } {\left( {\frac{8}{{{{(2n+1)}^2}{\pi ^2}}}} \right)} *{e^{\left( {\frac{{ - {D_g}{{(2n+1)}^2}{\pi ^2}t}}{{{L^2}}}} \right)}}$$

We modeled the diffusion of TXA, bupivacaine, tobramycin, and vancomycin from PEG diacrylate (PEGDA) hydrogels of varied molecular weights (575, 700, and 2000 g/mol), which are expected to have different mesh sizes (**Fig. ****S1**). Generally, the lower molecular weight of polymer used, the slower the diffusion of the therapeutic drug out of the hydrogel, due to the smaller calculated mesh size^36^. For therapeutics with molecular weights lower than ~ 500 g/mol, burst release from the network is expected^37^. Overall, the predicted release rates of the therapeutic agents from the hydrogels were faster than desired. However, cumulative release correlated with molecular size, with the largest molecule, vancomycin, diffusing and releasing the slowest. It is important to note that calculated mesh size can vary widely from actual mesh size, as many experimental parameters beyond the molecular weight of PEGDA can affect crosslink density of the hydrogel. (Note, bupivacaine was initially investigated in the predicted release computations, but we transitioned to lidocaine in the in vitro experiments due to its faster onset of action. It is similar in molecular size and chemical structure to bupivacaine, and thus its diffusion-based release is expected to be similar.)

### Mechanical characterization

Due to variations that can be seen between predicted and observed hydrogel mesh sizes, more accurate release profiles can be obtained by experimentally determining hydrogel mesh size. PEGDA of varying molecular weights was crosslinked using the redox initiating system of ammonium persulfate (APS) and tetramethylethylenediamine (TEMED), which produce free radicals when mixed in solution. Elastic moduli and swelling ratios can be used to estimate hydrogel mesh size, which determines diffusion rates of therapeutic agents (*Equation S6*, Table 1). Elastic moduli decrease as a function of increasing PEGDA molecular weight, which is consistent with the computational models. Conversely, mass swelling ratios (Q_m_) increase with PEGDA molecular weight. The Flory-Rehner theory was also used to calculate Q_v_ of hydrogels fabricated using different molecular weight PEGDA^38^. Q_m_, Q_v_, and mesh size increased as a function of PEGDA molecular weight. The elastic moduli, swelling ratios and mesh size, as well as the estimated hydrodynamic radii of each therapeutic agent, are summarized in Table 1.

**Table 1:** Swelling ratios and mesh size of hydrogels fabricated with different molecular weights of PEGDA.

PEGDA	Elastic modulus (kPa)	Q_m_	Q_v_	Mesh size (nm)	Therapeutic agent	*r*_s_ (nm)
700	519 ± 24.9	4.41 ± 0.06	4.88 ± 0.07	18.8 ± 0.09	Tranexamic acid	0.49
2000	382 ± 21.6	5.88 ± 0.04	6.95 ± 0.05	35.2 ± 0.09	Lidocaine	0.55
3350	175 ± 11.4	7.82 ± 0.09	9.25 ± 0.11	50.6 ± 0.20	Tobramycin	0.69
4600	118 ± 20.8	10.9 ± 0.35	12.9 ± 0.43	68.4 ± 0.76	Vancomycin	1.00

### Therapeutic release—drug release profiles and release efficacy

**Fig. 2:**
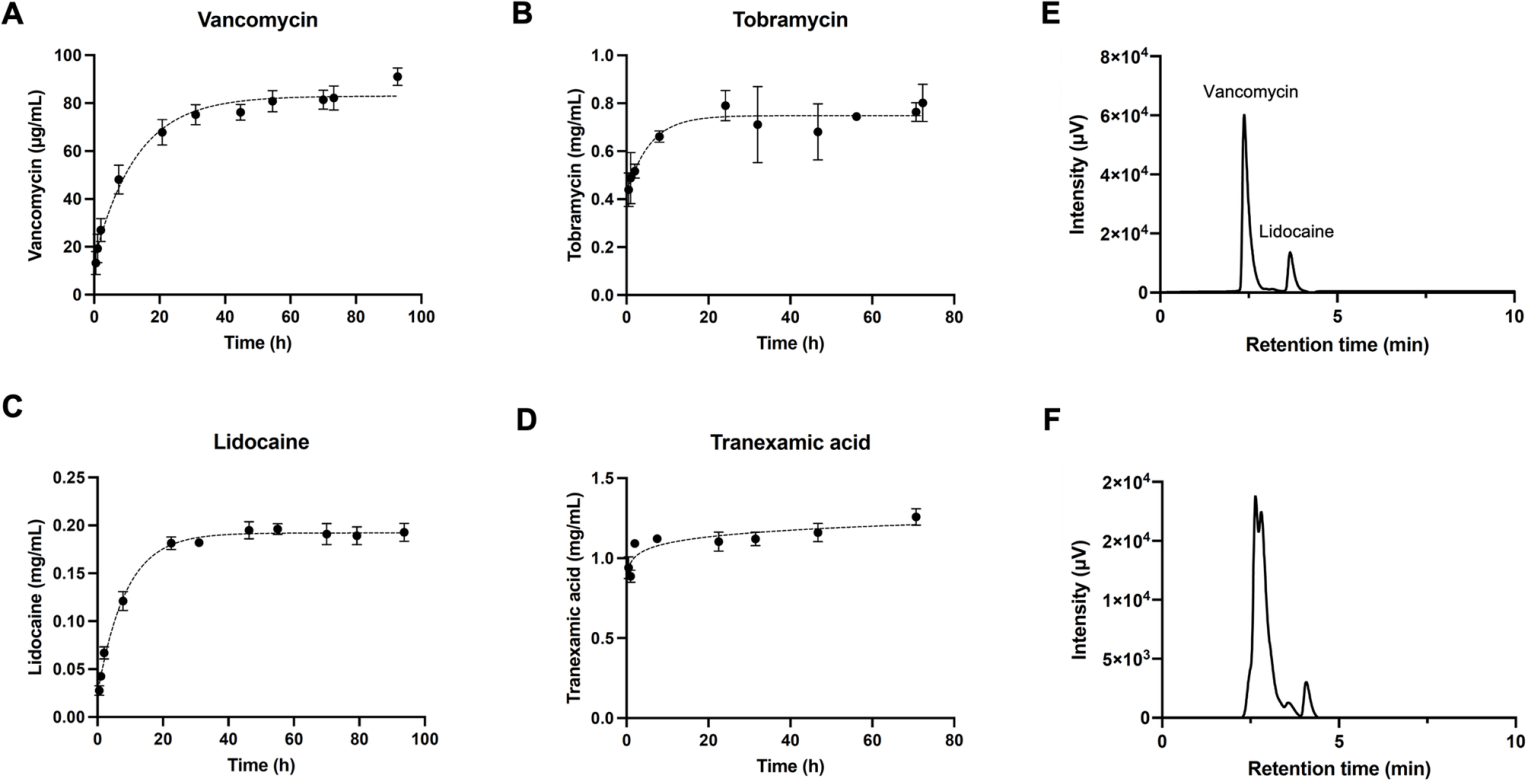
Kinetic release profiles of therapeutic agents from PEGDA 3350 hydrogels: (**A**) vancomycin, (**B**) tobramycin, (**C**) lidocaine, and (**D**) TXA. HPLC chromatograms (**E**) showing the retention times of vancomycin and lidocaine, and (**F**) confirming the release of vancomycin and lidocaine from a hydrogel system containing all therapeutics after 48 h in vitro elution. Retention times: Vancomycin; 2.6–3 min. Lidocaine; 3.7–4.2 min.

In vitro kinetic release profiles of hydrogels loaded with a single drug were quantified over time to assess release behavior (Fig. 2A-D). Vancomycin is slowly released over a four-day period, while tobramycin releases faster, with cumulative concentration plateauing after approximately 24 h. After 5 days, ~ 177.5 µg vancomycin and ~ 239.3 µg tobramycin had been released from hydrogel discs. The sustained release of antibiotics is desirable for extended treatment of bacterial challenges. Lidocaine diffuses out of the hydrogel over a 2.5-day period, with 80% release within 8 h. This balance of relatively quick initial release with continual release over several days is beneficial, as sufficient lidocaine will be released to provide pain relief quickly, but the intermediate duration of efficacy of lidocaine necessitates controlled relief for extended pain management. Finally, TXA exhibits burst release, which is ideal for controlling bleeding quickly.

HPLC and mass spectrometry were used to validate that multi-drug loading does not affect hydrogel release kinetics, using vancomycin as the model drug. Retention times for vancomycin and lidocaine were determined using a mixture of the therapeutic agents (Fig. 2E). HPLC was then performed on eluent from a hydrogel containing all therapeutic agents. These results indicate that 98% of vancomycin was released from the multi-drug loaded hydrogel after 48 h and confirm that multi-drug loading does not impact the release rates of therapeutic agents (Fig. 2F). Efficacy of our separation method was validated by our ESI-MS, which confirms the presence of vancomycin (**Fig. ****S2**).

### Efficacy of released antibiotics

A broth microdilution assay was performed using hydrogel eluent from antibiotic-loaded gels to determine the minimum inibitory concentration (MIC) for released antibiotics. Tobramycin and vancomycin were evaluated individually and together from a hydrogel loaded with both antibiotics (**Fig. S8**). Vancomycin released from the hydrogels had an MIC of 4.6 ± 1.5 µg/mL against *S. aureus*, and released tobramycin had an MIC of 7.5 ± 1.7 µg/mL against *E. coli* (Table 2). The MICs of dual-antibiotic-loaded hydrogels remained approximately unchanged. In vitro MIC testing was limited to *S. aureus* and *E. coli*, while antimicrobial efficacy against *P. aeruginosa* was evaluated exclusively in vivo using a polymicrobial murine open fracture model with longitudinal bioluminescent imaging and CFU quantification.

**Table 2:** MIC of antibiotics released from hydrogels against *S. aureus* and *E. coli*.

	MIC (CLSI/Lit.)	Tobramycin hydrogel	Vancomycin hydrogel	Tobramycin + vancomycin hydrogel
S. aureus	≤ 2 µg/mL	–	4.60 ± 1.50 µg/mL	3.60 ± 0.30 µg/mL
*E. coli*	≤ 1 µg/mL	7.48 ± 1.70 µg/mL	–	5.37 ± 0.50 µg/mL

### Efficacy of released TXA

**Fig. 3:**
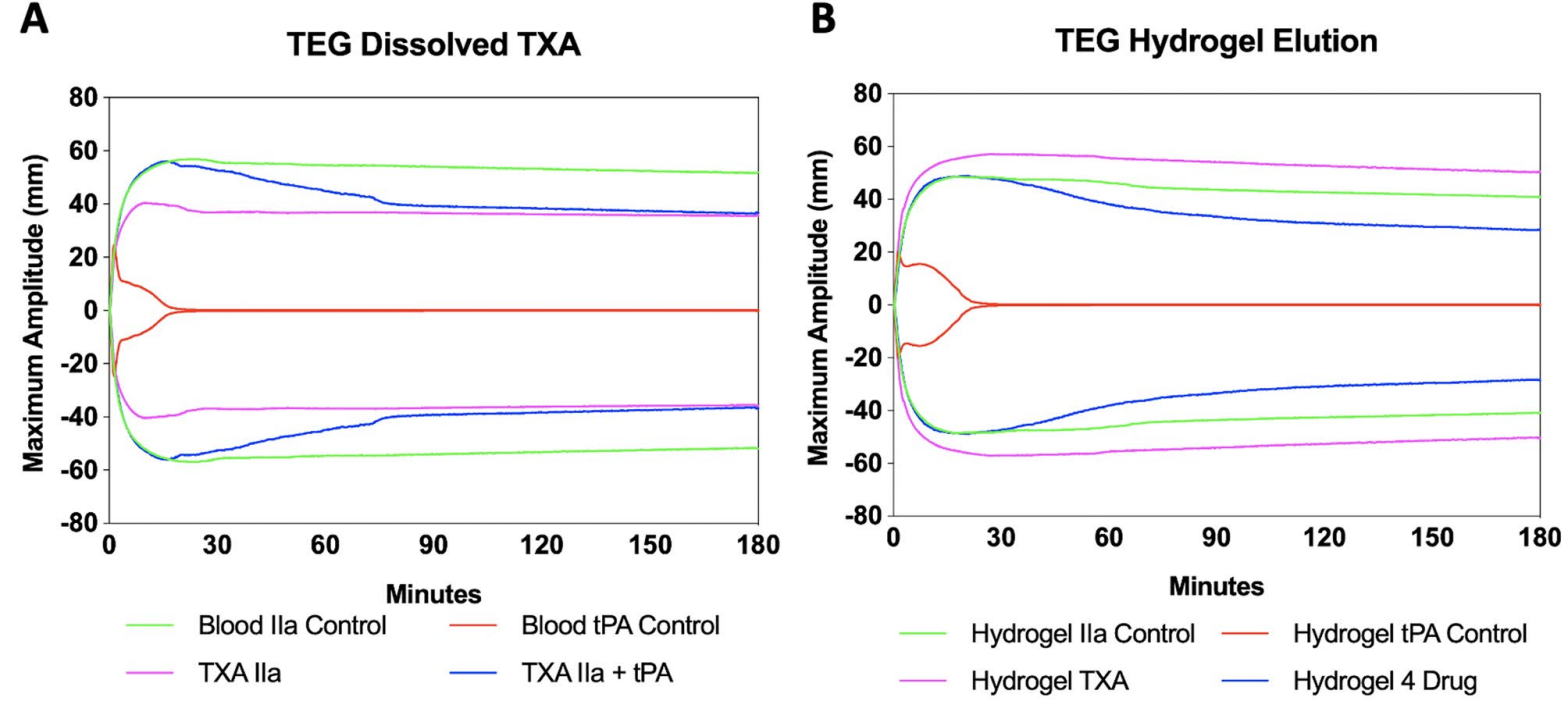
(**A**) TEG curve for experiment 1 testing C57BL/6 mouse blood with or without dissolved TXA. (**B**) TEG curve for experiment 2 demonstrating C57BL/6 mouse blood incubated with hydrogel overnight. Clot strength is determined by a measure of maximum amplitude (mm).

To ensure that the released TXA retained efficacy, we tested the ability of hydrogels loaded with TXA only and hydrogels loaded with all four therapeutics to prevent fibrinolysis in a thromboelastography (TEG) assay, an ex vivo test that quantifies the ability of whole blood to form a clot. Control experiments were performed to establish baseline performance for thrombin IIa, tPa, and TXA. In the IIa control group, a strong clot was successfully induced with maximum amplitude (MA), G, and clot lysis at 30 min (LY30) values (all indicators of clot strength) of 56.6 mm, 6.7 Pa, and 3.13%, respectively (Fig. 3A) In the tPA control group, we were able to induce fibrinolysis with MA, G, LY30 values outside of normal limits at 21.6 mm, 1.43 Pa, and 72.26%, respectively. For TXA IIa group, the addition of TXA did not disrupt our ability to induce clotting indicated by MA, G, and LY30 values of 56.8 mm, 6.76 Pa, and 4.36%, respectively. In the TXA + IIa + tPA group, dissolved TXA was able to prevent tPA fibrinolysis with MA, G, and LY30 values of 56.3 mm, 6.67 Pa, and 7.67%, respectively.

The addition of unloaded hydrogel did not induce coagulopathy indicated by MA, G, and LY30 values of 49 mm, 5 Pa, and 4.4%, respectively, which were similar in value to the controls. This blood also underwent successful fibrinolysis indicated by MA, G, and LY30 values of 27.1 mm, 1.97 Pa, and 84.6, respectively. TXA-impregnated hydrogels were able to prevent fibrinolysis indicated by MA, G, and LY30 values of 56.1 mm, 6.5 Pa, and 0.07, respectively. Lastly, 4 drug loaded hydrogel did not disrupt TXA elution and fibrinolysis was inhibited in the presence of tPA indicated by MA, G, and LY30 values of 49 mm, 5 Pa, and 4.4 respectively (Fig. 3B). Because TXA acts by inhibiting fibrinolysis rather than enhancing early clot formation or intrinsic clot strength, the modest reduction in MA and G observed with the 4 drug loaded hydrogel likely reflects formulation effects of additional therapeutics, while preserved suppression of LY30 confirms intact TXA antifibrinolytic activity. These experiments demonstrated that TXA impregnated and multi-drug impregnated hydrogels can successfully prevent fibrinolysis in a TEG assay, which suggests the efficacy of this therapy to decrease blood loss in the field.

### In vivo efficacy of hydrogel wound dressing

**Fig. 4:**
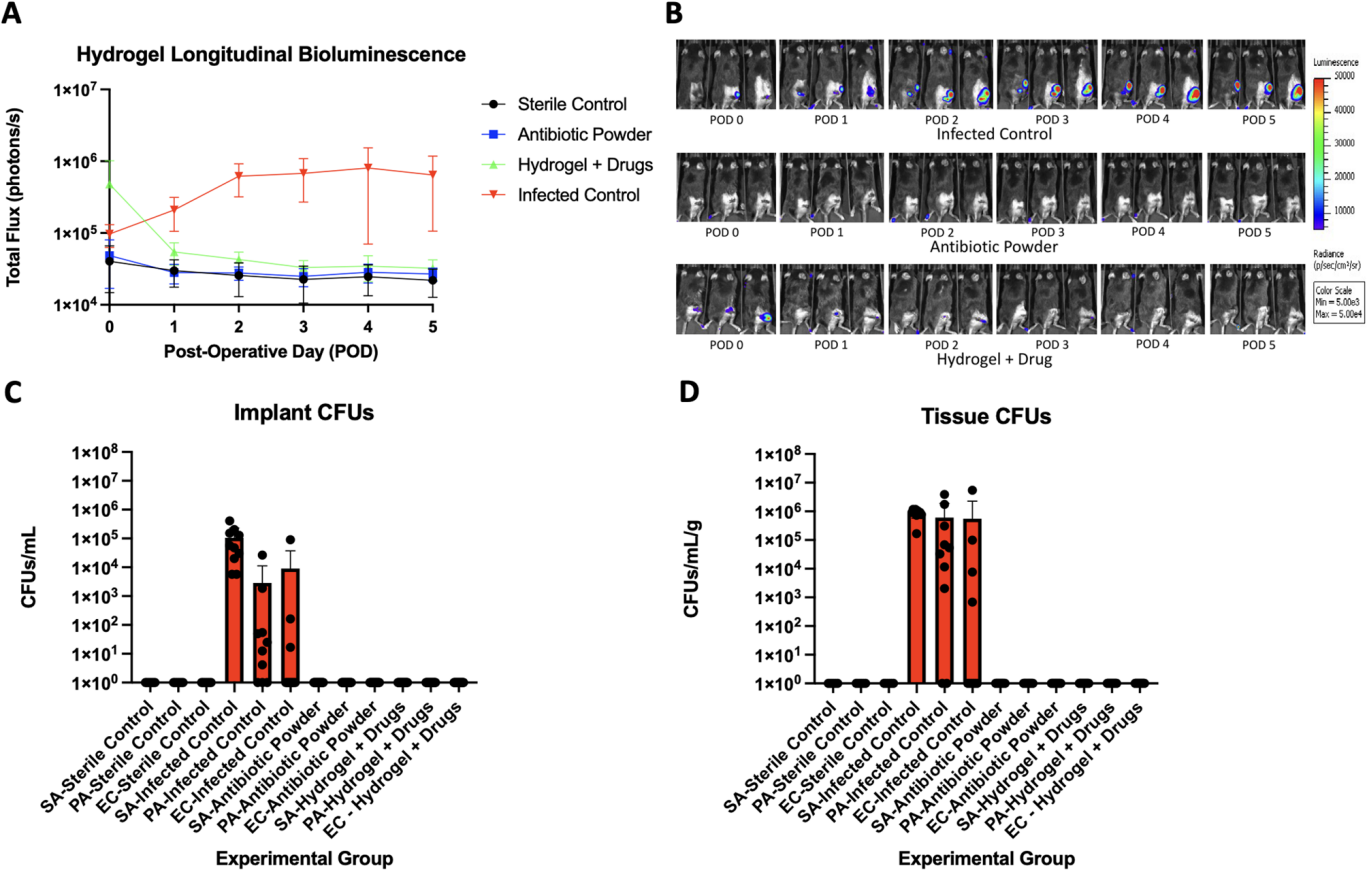
SC = sterile control. IC = infected control. AP = antibiotic powder. H + D = Hydrogel + drugs. (**A**) Longitudinal bioluminescence representing bacterial burden over 5 postoperative days. (**B**) Representative in vivo images demonstrating bioluminescent (bacterial) signal in representative mice from each experimental group on PODs 1, 3, and 5. (**C**) Implant CFUs from Xen36, Xen41, and Xen14 which were isolated and selectively grown per experimental group. (**D**) Tissue CFUs from Xen36, Xen41, and Xen14 which were isolated and selectively grown per experimental group.

To test the efficacy of drug loaded hydrogels in preventing infection, we applied them in murine open fractures inoculated with bioluminescent *S. aureus*, *E. coli*, and *P. aeruginosa* and tracked infectious burden in vivo via non-invasive bioluminescent imaging. Control mice were treated with antibiotic powder during surgery. In vivo longitudinal bioluminescence demonstrated that, as expected, the highest bioluminescent signal was seen in the infected control group (Fig. 4A and B). The drug loaded hydrogel group demonstrated a rapid decrease in bioluminescent signal over the first 24 h, with the residual signal reaching sterile control levels after 72 h, indicating the consistent elution of antibiotics over this period. The antibiotic powder group demonstrated no bioluminescent signal throughout the post-operative period. Of the thirty mice who received either antibiotic powder or drug loaded hydrogel, all mice were culture negative at the end of the experiment (Fig. 4C and D). Following this experiment, the hydrogels were explanted from the murine wound model and underwent three extraction cycles at post-operative day (POD)5. Subsequent analysis via HPLC and mass spectrometry confirmed the absence of residual therapeutics entrapped within the hydrogel matrix.

### Tissue histologic analysis

**Fig. 5:**
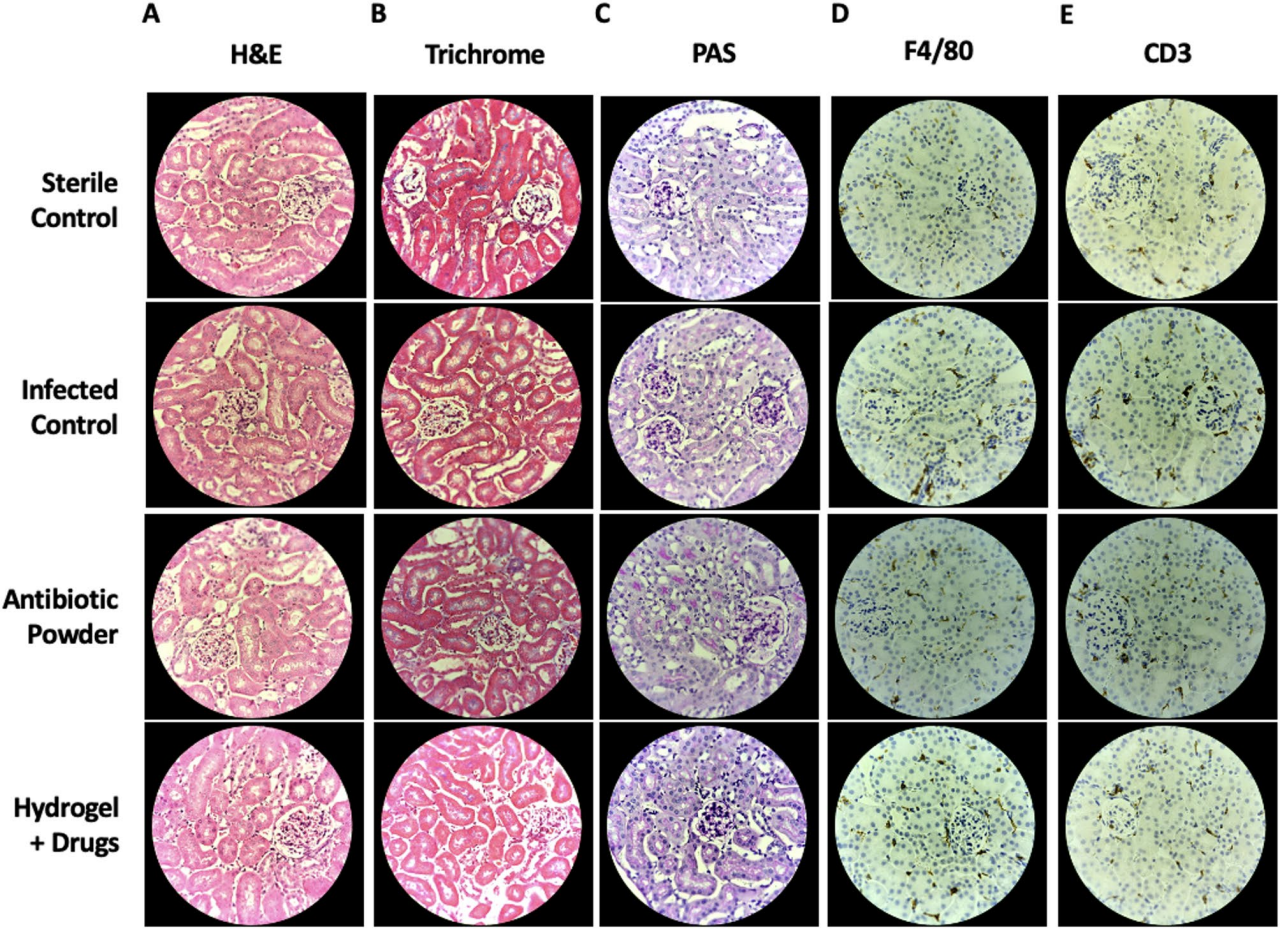
Kidney sections obtained per experimental group on POD5 (**A**) H&E (**B**) Trichrome (**C**) PAS (**D**) IHC F4/80 (**E**) IHC CD3.

Renal tissue sections were reviewed for anatomic abnormalities across all samples. There were no morphologic alterations of glomeruli and the tubulointerstitium on H&E sections (Fig. 5A). Basements membranes were intact in all samples when reviewing PAS-stained sections (Fig. 5B). No increased collagen deposition was noted on trichrome stained samples. (Fig. 5C). No innate (macrophage) or adaptive (T-cell) immune infiltrates were noted within the kidney tissue in all samples (Fig. 5D and E). These findings are pertinent given that vancomycin, tobramycin, and TXA are almost exclusively eliminated via the kidneys, demonstrating the safe loading and release of drugs from hydrogel wound dressing.

### Merino sheep wound model

**Fig. 6:**
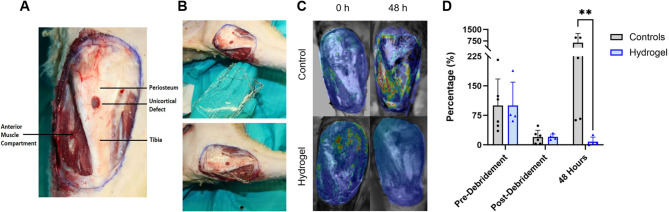
(**A**) Merino sheep complex musculoskeletal wound model, with injuries to the anterior tibia, muscle, fascia, and periosteum. (**B**) Hydrogel application in sheep model (**C**) Images of bioluminescent bacteria within the wound 0 h and 48 h post-debridement (**D**) *S. aureus* contaminated wounds were created in the ovine model and underwent debridement after 6 h. Photon counts were normalized with respect to the pre-debridement values of each respective group, for percentage of pre-debridement bacterial burden.

For our large animal studies, an open fracture and complex wound of the proximal tibia was created in adult Merino female sheep and inoculated with bioluminescent *S. aureus* Xen36 spread over the entirety of the wound surface (Fig. 6A). After a 6 h incubation, sheep underwent surgical wound debridement, and wound dressings were polymerized and subsequently applied (Fig. 6B). Control animals did not receive any treatment beyond debridement. The control- and hydrogel-treated groups were imaged with a photon-counting camera 0 h post-debridement and 48 h post-debridement (Fig. 6C). After 48 h, the control group (*grey*) exhibited a *632.1%* ± 602.6% increase in *S. aureus* burden relative to pre-debridement levels (Fig. 6D), while the hydrogel-treated group (*blue*) showed a *92% reduction* (from 100% to 8.1%), highlighting the hydrogel’s antimicrobial efficacy in large, complex wounds. The large standard deviation of the hydrogel group is due to sheep #4, in which the hydrogel had slipped out of place; even in this instance, the bacterial burden was 25.8% relative to pre-debridement levels. Were this animal participant excluded from analysis, the hydrogel-treated group exhibits a burden of 2.3 ± 2.0%. These data suggest that these therapeutic releasing hydrogels can prevent infection and sepsis for at least 48 h post-traumatic injury.

## Discussion

Scenarios with limited medical resources, such as remote trauma, wilderness injuries, mass casualty events, or natural disasters, require innovative solutions for managing wounds outside of traditional healthcare infrastructure. This work aimed to develop a field-polymerizable hydrogel wound dressing capable of delivering antimicrobial, hemostatic, and analgesic agents directly at the point of injury using only potable water, without the need for specialized equipment or storage conditions.

Using computational modeling, we optimized the properties of a PEG-based hydrogel to achieve rapid polymerization, robust structural integrity, and controlled therapeutic release^39^. PEG 3350 (MiraLax^®^) was selected as the starting material for acrylation, due to its established safety profile, long-term stability, and regulatory familiarity—factors that streamline translation^40,41^. The PEGDA powder remained stable at elevated temperatures (46 °C) for at least 3 months, an important consideration for storage and transport in rural or disaster-prone settings. APS, a stable solid, can be pre-mixed with PEGDA and therapeutics, while TEMED—liquid and volatile at room temperature—requires airtight packaging and separate addition with water to activate the system (*Supplemental Methods S2. Stability studies)*. We are also investigating alternatives to TEMED that are less volatile and more cytocompatible (DOI: 10.1021/acsabm.3c01264).

This platform demonstrated controlled release of vancomycin and tobramycin over several days, addressing both immediate and sustained infection control. The hydrogel platform was engineered to locally deliver tranexamic acid (TXA) and lidocaine, with in vitro release kinetics and preserved antifibrinolytic activity supporting its intended roles in hemorrhage mitigation and localized pain management in traumatic wounds. The hydrogel formed within two minutes at room temperature and showed favorable swelling and mechanical properties to absorb wound exudate and conform to tissue architecture.

Importantly, antibiotic-loaded hydrogels retained antimicrobial efficacy against *S. aureus*, *E. coli*, and *P. aeruginosa* both in vitro and in vivo, including a rigorous polymicrobial open fracture mouse model^42^. The inclusion of local lidocaine delivery highlights the potential to reduce reliance on systemic opioids, which carry risks of respiratory depression and abuse.

The TXA-impregnated hydrogels prevented fibrinolysis in thromboelastography (TEG) assays and preserved clot strength, confirming the biological activity of the eluted drug. Importantly, kidney histology revealed no morphologic or inflammatory changes across treatment groups, supporting the renal safety profile of the eluted agents and the hydrogel itself.

In a large animal model, the hydrogel dressing significantly reduced bacterial burden in complex contaminated wounds, demonstrating its translational potential in larger tissue volumes and irregular wound beds. These findings suggest this hydrogel could reduce the risk of biofilm formation and sepsis particularly when immediate surgical debridement or hospital transfer is not possible.

The modularity of this platform allows for customization of therapeutic payloads, enabling application beyond open fractures such as in burns, chronic wounds, or fungal skin infections by substituting or adding relevant agents. Its powder formulation, field-stability, and rapid polymerization position it as a highly adaptable solution for prehospital care, humanitarian response, wilderness medicine, and rural health delivery systems.

While our findings demonstrate robust antimicrobial and hemostatic efficacy across small and large animal models, several limitations remain. This platform has not yet been evaluated in human subjects, and additional studies will be necessary to assess safety, tolerability, and clinical usability. From a regulatory perspective, the hydrogel may be considered a drug delivery system, as its primary function is to locally deliver FDA approved therapeutics in a controlled manner. This classification may streamline the translational process by leveraging existing safety data and avoiding the need for novel therapeutic approvals. While our current results support short term efficacy, longer term studies are warranted to evaluate tissue integration, biodegradation, and healing outcomes. Future work will focus on expanded large animal testing in contaminated musculoskeletal wounds and on assessment under simulated or real-world field conditions to evaluate polymerization kinetics, durability, and user operability. These efforts will inform potential deployment for military medicine, wilderness rescue, disaster response, and rural trauma care.

## Materials and methods

### Materials

Polyethylene glycol 3350 g/mol (Integra Chemicals), acryloyl chloride (Sigma, *≥* 97%), triethylamine (Sigma, *≥* 99%), dichloromethane (Fisher Scientific, *≥* 99.5%), potassium carbonate (K_2_CO_3_, Fisher Scientific, 99.3%), anhydrous magnesium sulfate (MgSO_4_, Fisher Scientific), ether (Fisher Scientific, 99%), deuterated chloroform (Fisher Scientific, 99.8%), ammonium persulfate (APS, Amresco, ACS Grade), tetramethylethylenediamine (TEMED, Amresco, *≥* 99%), vancomycin (Fisher Scientific, ≥ 80%), lidocaine (Fisher Scientific), tranexamic acid (Fisher Scientific, ≥ 98%), tobramycin (Fisher Scientific, ≥ 94%), LB broth, Miller (Fisher BioReagents), phosphate buffered saline, PBS (VWR), acetonitrile, HPLC grade (Fisher scientific, 99.95%), water, HPLC grade (Fisher scientific), trifluoroacetic acid, TFA (Avantor, 99.5%), Bleach (Fisher scientific, 8.25%), Histoprep Ethanol (Fisher Scientific, 70%), Phosphate Buffered Formalin (Fisher Scientific, 10%), Phosphate Buffered Saline pH 7.4 (Fisher Scientific), Tryptic Soy Broth (BD Bacto), Luria Broth (BD Bacto), Dehydrated Agar (BD Bacto), Kanamycin (Fisher Scientific, *≥* 99%), Tetracycline (Fisher Scientific, *≥* 95%), Thrombin (Millipore sigma), tPA (Millipore sigma), Vacutainer tubes with Sodium Citrate (BD, 3.2%), Micro blood collecting tube with ammonium heparin (Fisher Scientific), Buprenorphine SR (Ethiqa), Distilled water (Fisher Scientific), 0.8 mm titanium Kirschner wires (Depuy Synthes), IVISbrite *Escherichia coli* Xen14 (Revvity), IVISbrite *Staphylococcus aureus* Xen36 (Revvity), IVISbrite *Pseudomonas aeruginosa* Xen41 (Revvity), Mixed Merino sheep; 39.5 kg ± 4.0 (Josh Talley Ranch, 3936 FM 1052 Uvalde, TX 78801), ketamine (KetaVed™; Phoenix Scientific, St. Joseph, MO), isoflurane (Piramal Healthcare Ltd. India), buprenorphine (Buprenorphine SR™, ZooPharm Laramie, WY),

### Study design

The first phase of investigation in this study focused on computational modeling used to predict drug diffusion out of various PEGDA hydrogel systems to narrow down candidates (*Supplemental Methods S1. Computational Modeling*). We then experimentally investigated and optimized the in vitro release profile of tranexamic acid (TXA), lidocaine, tobramycin, and vancomycin from the hydrogels, characterized their mechanical properties and stability, and evaluated blood clotting time. Once optimized, antimicrobial efficacy and hemostasis were extensively tested in our murine model of open fracture. Upon proving a track record of efficacy, the hydrogel was tested in a cohort of Merino sheep. Animals were randomly assigned to control and experimental groups. Sample sizes were determined via power analysis and are representative of our prior studies.

### Synthesis of polyethylene glycol-diacrylate

Polyethylene glycol-diacrylate (PEGDA) was synthesized using previously established methods (**Fig. ****S3**)^43,44^. The purification of the macromer was slightly modified, washing with 2 M K_2_CO_3_, drying the organic layer over MgSO_4,_ and concentrating by rotary evaporation. The product was precipitated into chilled ether and collected via filtration. The precipitate was dried overnight by reduced pressure to yield a white powder (6.92 g, 80.1%). Each batch was individually tested to ensure at least 75% conversion and a gelation time of ≤ 2 min at room temperature. ^1^H NMR (CDCl_3_, ppm): δ = 6.50 (d, 2 H), 6.20 (m, 2 H), 5.82 (d, 2 H), 4.25 (t, 4 H), 3.70–3.55 (m, 320 H) (**Fig. S4**). ^1^H NMR was also used to confirm the stability of PEGDA 3350 after 3-month storage at elevated temperatures (*Supplemental Methods S2*, **Fig. S5**).

### PEGDA hydrogels

Final concentrations of PEGDA, APS, and TEMED were kept consistent for in vitro and in vivo experiments. For all hydrogels, final reagent concentrations were: 24.14 wt% PEGDA, 30 mM APS, and 30 mM TEMED. PEGDA was diluted to 35.5 wt% in DI H_2_O and vortexed until dissolved. TEMED and APS were diluted to 1 M, prepared fresh prior to each use. To prepare 1 mL hydrogels, 680 µL 35.5 wt% PEGDA and 260 µL DI H_2_O or drug stock solution (*Supplemental Methods S3. Drug Stock Solutions*) were combined in an Eppendorf tube. To initiate crosslinking, 30 µL 1 M TEMED and 30 µL 1 M APS were added, pipette mixed, and immediately used. For murine and large animal experiments, hydrogel volumes were changed to 300 µL and 35 mL respectively.

### Computational modelling and therapeutic release kinetics

Computational modeling was used to provide insight into hydrogel properties and therefore predict the release kinetics of therapeutic agents^22,36^. Hydrogels were modeled as thin films, allowing therapeutic drug concentration as a function of time to be calculated (Eq. 1)^22^.

The entire model is discussed in detail in *Supplemental Information Section S1*. For in vitro experiments, the experimental workflow is depicted in **Fig. S6**. The pre-gel solution was pipetted onto a glass slide with 2 mm spacers, and a glass slide was placed on top. After gelation, a 6 mm biopsy punch was used to make uniform hydrogel sample discs, which were transferred to 1 mL DI H_2_O in a 48 well plate. Each time point included one blank PEGDA hydrogel disc and three drug-loaded hydrogel discs in separate wells.

At each time point, the absorbance of the eluent from the drug-loaded hydrogels was measured using a UV-Vis spectrometer (Biomate 3 S, Thermo Scientific – Waltham, MA, USA), with the unloaded hydrogels serving as the blank. Calibration curves of each drug were used to relate absorbance to concentration, allowing the amount of drug released at each time point to be determined (*Supplemental Methods S4*, **Fig. S7**).

Tobramycin and tranexamic acid lack UV absorbing chromophores, necessitating alternative detection approaches. A ferric chloride assay was used to detect tobramycin and tranexamic acid, and an additional copper sulfate assay was developed to quantify tobramycin at high concentrations. While this ferric chloride assay has previously been reported to detect tranexamic acid, to our knowledge, this is its first reported use for the detection of tobramycin^45^.

### Efficacy of released antibiotics

A broth microdilution assay was performed to validate that antibiotics released from the hydrogel system retained their antimicrobial activity. Drug-free, vancomycin-loaded (V), tobramycin-loaded (T), and vancomycin+tobramycin-loaded (V + T) PEGDA 3350 hydrogels were prepared as previously described.

The sample discs were incubated at room temperature in 500 µL PBS for 5 days. UV-Vis spectrometry was used to determine the concentration of vancomycin and tobramycin released from each hydrogel disc. The eluent was then used in an LB broth microdilution assay with *E. coli (MG1655)* and *S. aureus (SA113)* at burdens of 5E5 CFU/mL, with sterile media controls and growth controls (**Fig. S8**)^46^. After incubating at 37 °C for 18 h, the plates were examined to determine the MIC. The MICs of tobramycin and vancomycin released from hydrogels containing each antibiotic alone were quantified for *E. coli* and *S. aureus*, respectively. These were then compared to the MICs of antibiotics released from the dual-antibiotic-loaded hydrogels.

### Efficacy of released TXA

The effect of the presence of TXA with and without the hydrogel on mouse whole blood clot lysis was evaluated with thromboelastography (TEG) using a TEG 5000 (Haemonetics Corp, Braintree, MA, USA). Briefly, thrombin (IIa, alpha-thrombin) was used to initiate clot formation in whole blood and fibrinolysis was induced by simultaneous addition of tPA. Each clot formation/lysis assay contained 300 µL of citrated mouse whole blood, alpha-thrombin (6 µg/mL), tPA (3.3 µg/mL), CaCl_2_ (10 mM) and 200 μm dissolved TXA or up to 0.477 µM of eluted TXA from hydrogel in TBS/BSA (50 mM Tris-HCl, containing 100 mM NaCl and 0.1 mg/mL bovine serum albumin) solution to make the final volume to 360 µL. A 56.5uL uniform hydrogel cylinder was combined with 2 mLs of blood in sodium citrate containers and incubated overnight for experiments involving hydrogel elution. The tPA concentration was chosen based on the generated plasmin effect on the clot strength and lysis. Each experiment was performed for 180 min to establish the LY60 values. The thromboelastograph was calibrated each day, and each experiment was tested in triplicate. TEG Analytical Software (Version 4.2.2; Haemonetics Corporation, Braintree, MA, USA) was used to calculate maximal clot strength (maximal amplitude (MA), which was directly related to the shear elastic modulus strength, G), and percent lysis 60 min after MA (LY60).

### HPLC and mass spectrometry

To investigate the release of multiple therapeutics from our hydrogel system, we employed chromatographic techniques and spectrometric analysis. The chromatographic system is described in *Supplemental Methods S5*, with calibration curves shown in **Figure S9**. The separation of the therapeutics were achieved using a 4.6 × 250 mm, 5 μm particle size, C18 column. The mobile phase consisted of a 70:30 (v/v) isocratic mixture of HPLC-grade water and acetonitrile + 0.1% trifluoroacetic acid. The flow rate was set at 0.5 mL/min and the UV detection wavelength was set at 240 nm (an average between UV_max_ of vancomycin and lidocaine). The injection volume was 20 µL with a total run time of 15 min^47–49^.

For mass spectrometry analysis, the samples were infused using a direct-loop injection on a Waters Acquity UPLC system, separated using an Acquity BEH 50 × 2.1 mm, 1.7 μm particle size C18 column, and eluted with a gradient of 3–95% solvent A and B over 15 min (solvent A: water, solvent B: acetonitrile, with 0.3% and 0.2% formic acid respectively (v/v)). Mass spectra were recorded from a mass of 300–2000 Da. To ensure accuracy and optimal calibration of these methods, standard curves for each therapeutic were plotted.

### Characterization of hydrogels

Mechanical characterization was performed on hydrogels fabricated using a range of PEGDA molecular weights to evaluate elastic moduli and swelling ratios. Mass swelling ratios were calculated by taking the ratio of swollen hydrogel mass to the dry hydrogel mass following lyophilization. A dynamic mechanical analyzer (Q-800, TA Instruments – New Castle, DE, USA) was used to measure the elastic modulus of hydrogels. Using a uniaxial compression test, the initial preload force was set to 0.001 N and the initial strain was set to 0.5%, and the system ramped the strain 2.5%/min to a maximum strain of -20%. The linear region of the stress-strain curve was used to calculate elastic modulus.

### In vivo experiments

#### Preparation of murine inoculum

Methods were derived and modified from our previous work^43^. Bioluminescent strains of methicillin-sensitive *S. aureus* ATCC 49,525 (Xen36), *E. coli* WS2572 (Xen14), and *P. aeruginosa* PAO1 (Xen41), containing optimized photorhabdus luminescens lux operons, were streaked on LB 1.5% agar plates with 200 µg/mL kanamycin, 30 µg/mL kanamycin, and 60 µg/mL tetracycline, respectively (Revvity). Use of bioluminescent strains enables infectious burdens to be longitudinally monitored in real time throughout the course of the experiment. Overnight cultures were prepared as previously described after which the bacterial cells were pelleted, resuspended, and washed three times in PBS. Final inoculums *S. aureus*,* E. coli*,* and P. aeruginosa* of 1E5, 1E3, and 1E2 colony forming units (CFUs), respectively, in 2 µL PBS were estimated by measuring the absorbance at 600 nm (A600; Biomate 3; Thermo). Inocula were placed on ice to prevent bacterial death. CFUs were counted and verified after overnight incubation of plated inoculum at the end of surgery.

### Ethics statement

All animal experiments were approved by the University of California, Los Angeles (UCLA) Institutional Animal Care and Use Committee (IACUC) for murine studies (ARC# 2008 − 112) and by the University of Texas Medical Branch (UTMB) Institutional Animal Care and Use Committee for ovine studies (ARC# 2003030 A). All procedures were carried out in accordance with institutional guidelines and the National Institutes of Health Guide for the Care and Use of Laboratory Animals. All methods are reported in accordance with the ARRIVE guidelines.

### Study design, randomization, blinding, and sample size determination

Animals were randomly assigned to treatment groups. Investigators were not blinded to group allocation during outcome assessment, as the procedural and postoperative handling requirements made blinding impractical in this model. Sample size was determined a priori based on previously published musculoskeletal infection models, targeting the ability to detect at least a one-log reduction in CFU burden with 80% power at an alpha of 0.05. Inclusion criteria consisted of animals that completed surgery without intraoperative complication. Predefined exclusion criteria included early postoperative mortality, hardware failure, development of lameness, or reaching a humane endpoint due to severe infection or systemic illness.

#### Open fracture murine model

**Fig. 7:**
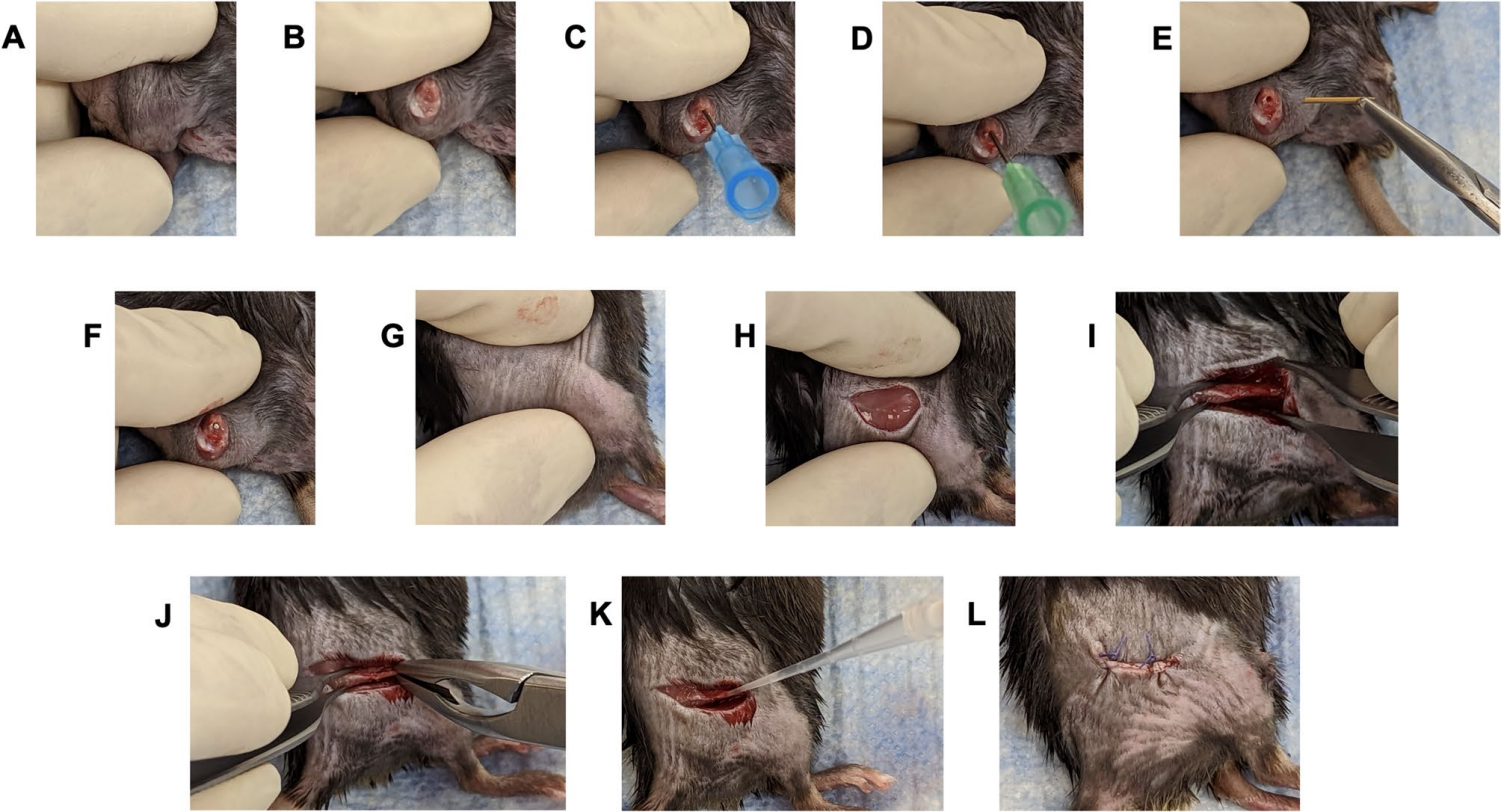
(**A** & **B**) The joint is exposed and a medial parapatellar arthrotomy is made. (**C** & **D**) The femur is sequentially reamed using 25 g and 21 g needles. (**E** & **F**) The titanium implant is inserted retrograde into the intramedullary canal. (**G**) The mouse is positioned in lateral decubitus and the second incision is made 1 cm proximal to the joint space. (**H**) A lateral approach to the femur is performed. (**I**) Adson forceps are used to bluntly dissect the anterior and posterior soft tissue compartments and the femur is exposed. (**J**) A 2 mm lateral cortical defect is created using a rongeur along the distal femoral meta-diaphysis. (**K**) The lateral cortical defect is inoculated with a bacterial suspension. (**L**) The skin incision is closed using two horizontal mattress sutures.

Twelve-week-old C57BL/6 mice were utilized for this model. Animals were housed in a temperature- and humidity-controlled barrier facility with ad libitum access to food and water on a 12-hour light–dark cycle. They were assessed daily by veterinary staff and lab members to ensure their health. This surgical procedure was adapted from our previously described open fracture model with some modifications^50^. All murine procedures were performed under inhalational anesthesia using 2.5% isoflurane in oxygen delivered via a nose cone. Mice are treated with sustained-release Buprenorphine (2.5 mg/kg) pre-operatively and 72 h post-operatively. Each injection affords 72 h of analgesic coverage. Mice weighed approximately 25 g at the time of surgery. At the experimental endpoint, euthanasia was performed under deep isoflurane anesthesia followed by cervical dislocation, consistent with American Veterinary Medical Association (AVMA) guidelines. While the mouse is supine, a medial parapatellar arthrotomy is performed exposing the distal femur. The superior aspect of the intercondylar fossa is identified as the start point and sequentially reamed with 25 g and 21 g needles. A more superior start point matches the bow of the femur and allows for the placement of the longer implant utilized in this model. A 0.8 mm x 10 mm titanium Kirschner wire (Depuy Synthes) is placed retrograde into the intramedullary canal. The patella is reduced over the distal femur and incision closed using a horizontal mattress stitch using absorbable 3 − 0 Vicryl suture (Ethibond). The mouse is then positioned in the lateral decubitus position and a 10 mm long incision is made along the lateral aspect of the thigh directly over the femur. The vastus lateralis is split and overlying anterior and posterior compartment muscles bluntly dissected off the femur. A micro-rongeur is utilized to generate a 2 mm lateral cortical defect along the distal femur. The cortical defect is inoculated with a micropipette with E5 CFUs of Xen36, E2 CFUs of Xen41, and E3 CFUs of Xen14. Hydrogels were prepared as previously described, using the drug concentrations outlined in *Supplemental Methods S3.* The 300 µL pre-gel solution was pipetted into the tissue bed prior to gelation and allowed 90–120 s to solidify prior to skin closure. Once solidified, the incision was closed with 2 horizontal mattress stitches (Fig. 7).

### Quantification of real-time bacterial burden in vivo utilizing longitudinal bioluminescence

Methods were derived from our previous work^51^. Mice were imaged on postoperative days 0–5. Mice were anesthetized via 2.5% isoflurane inhalation and bioluminescence imaging was performed using a preclinical optical imaging system (IVIS Spectrum; Revvity). A maximum of three mice were positioned prone atop the system’s heated platform and tilted slightly to maximize exposure of the lateral operative thigh. Bioluminescent data could be acquired in sequence within the same acquisition. Mice were imaged using the following bioluminescent acquisition settings: field of view B, large binning, F/Stop 2, open filter, and 1 min acquisition. Data are presented on color scale overlaid on a grayscale photograph of mice, with bioluminescence quantified as total flux (photons/s/cm^2^) within a circular region of interest (1E3 pixels) using Living Image software (Revvity).

### Ex vivo quantification of colony forming units (CFUs)

Implant and tissue burden was quantified on POD5 at the termination of the experiment and protocols are slightly modified from prior work^51^. Implant CFUs were quantified by detaching adherent bacteria via serial vortexing and sonication in 500 uL of 0.3% Tween-20 in tryptic soy broth (TSB) for 2 and 10 min, respectively. In addition, bone and surrounding soft tissue were homogenized to quantify tissue burden of infection (Pro200^®^ Series homogenizer; Pro Scientific). Given the presence of multiple bacteria of interest, selective media was utilized to isolate and quantify bacteria. *S. aureus* was isolated by creating plates containing TSB, 1.5% bacto-agar, 7.5% NaCl. *P. aeruginosa* was isolated by creating plates containing LB, 1.5% bacto-agar, 2 ug/ml vancomycin, and 100 ug/ml ampicillin. *E. coli* was isolated by creating plates containing TSB, 1.5% bacto-agar, 2 ug/ml vancomycin, and 20 ug/ml ceftazidime. Implant and tissue CFUs were determined by counting CFUs after overnight incubation of implant sonicate and tissue homogenate on plates. Implant CFUs were expressed as CFUs/mL. Tissue CFUs were expressed as CFUs/mL/g.

### Tissue histologic analysis

On POD5 at experimental endpoint, mouse kidneys were harvested. Organs were bisected in the sagittal plane and placed in 10% phosphate buffered formalin for 24 h (Fisher Scientific). Cassettes were subsequently removed, washed with distilled water, and placed in 70% molecular ethanol at 4℃ (Fisher Scientific). Cassettes were delivered to our institution’s Translational Pathology Core Laboratory (TPCL) who embedded tissues in paraffin blocks and performed mounting on slides. Slides were stained separately with hematoxylin & eosin (H&E), periodic acid-Schiff (PAS), and trichrome stains. In addition, immunohistochemistry (IHC) was also performed to label F4/80, a marker for macrophages, and CD3, a general T-cell marker. Slides were reviewed for any pathologic abnormalities by a fellowship trained renal pathologist.

### Merino sheep wound model

For ovine procedures, animals were sedated with ketamine and midazolam, intubated, and maintained under general anesthesia with isoflurane and supplemental oxygen. An epidural injection of morphine (0.1 mg/kg diluted in sterile saline to a volume of 0.13 mL/kg) was administered to augment anesthesia and provide durable postoperative analgesia. The sheep were transported to the operating room and positioned supine on the operating table. At the predetermined study endpoint, sheep were euthanized with an intravenous overdose of pentobarbital solution administered under general anesthesia, in compliance with AVMA euthanasia guidelines. Using previously described methods, an open fracture and complex wound of the proximal tibia was created under deep anesthesia in the right hind limb of ten adult Merino female sheep (Average weight 39.5 ± 1.3 kg). Briefly, a 35-cm^2^ rectangular section of the skin and fascia overlying the proximal anterior tibia were removed followed by stripping of periosteum and creation of a 10-mm unicortical defect in the metaphysis of the proximal tibia. Using bovie electrocautery thirteen grams of muscle was removed from the tibialis anterior followed by a freeze injury (causing deep tissue damage), then a thermal burn injury was performed via bovie electrocautery to all remaining musculature, fascia, and periosteum (Fig. 6A)^52^.

The wound was then inoculated with 1 ml of 10^8^ colony-forming units/mL of bioluminescent *Staphylococcus aureus* (Xen36, Revvity, Massachusetts, USA) spread over the entirety of the wound surface. The wounds were subsequently bandaged using 4 × 4 gauze pads and wrapped with Kerlix dressings, the animals were then recovered with continued analgesia and allowed activity as desired^52^.

At 6 h following the inoculation of the open fracture wound the sheep were re-anaesthetized and placed into a light-free chamber. Then, the photon-counting camera (ORCA-Fusion-BT Digital Camera, Hamamatsu Photonics, Inc., Hamamatsu-City, Japan) was used to quantify and determine the local densities of viable bacteria within the wound bed. Immediately following the establishment of a baseline level of bacteria, sharp debridement and irrigation of the wounds were performed with 3 L of normal saline using gravity flow irrigation. After irrigation and debridement, the imaging sequence was repeated to quantify bacteria levels post-debridement. The sheep were then assigned into either the control or the hydrogel-treated group and re-imaged 48 h after injury. 35mL of pre-gel hydrogel was cast in mold sterilized with ethanol. Following gelation, the gel was placed on the wound and held in placed by surgical gauze and compression bandages. The hydrogel concentrations and drug stock solutions (*Supplemental Methods S3. Drug Stock Solutions*) for this model remained constant with previous experiment and were scaled up as required.

### Statistical analysis

Statistical testing and data visualization were performed using Prism (GraphPad). All animals that participated in this study were included in analyses. Statistical significance was established using two-tailed unpaired Student’s *t* tests with a threshold of *P* < 0.05.

## Supplementary Information

Below is the link to the electronic supplementary material.

Supplementary Material 1

Supplementary Material 2

Supplementary Material 3

## Data Availability

The data supporting the findings of this study are available within the article and its Supplementary Information files, including all computational modeling parameters, experimental methods, release profiles, and supporting figures in the Supplemental Materials document. Additional raw data files, including drug-release datasets, MIC assay outputs, chromatogram exports, and imaging data, are available from the corresponding author upon reasonable request.
