# Supplementary material for: A field polymerizing hydrogel enables simultaneous antimicrobial, hemostatic, and analgesic delivery in traumatic wounds
Source: Sci Rep. 2026 Feb 2;16:6950. doi: 10.1038/s41598-026-37521-y (PMC12917186; doi:10.1038/s41598-026-37521-y)
Supplement: Supplementary file 1 — Supplementary Material 1 [file 41598_2026_37521_MOESM1_ESM.pdf]

Title: A Field-Polymerizable Hydrogel for Simultaneous Antimicrobial, Hemostatic, and Analgesic Delivery in Traumatic Wounds

One Sentence Summary: Conceptualization, design, and construction of an antimicrobial, hemostatic, and analgesic drug-eluting hydrogel wound dressing for use in traumatic injuries in remote environments, validated with preclinical testing in small and large animals.

Elizabeth A Pumford[*1], Christopher D Hamad[*2], Amaka I Enueme[1], Zeinab Mamouei[2], Nicholas Peterson[2], Christopher Hart[2], Chad Ishmael[2], Alan Li[3], Rahul Sobti[4], Jack Pearce[4], Jeremiah Taylor[3], Micah Ralston[4], Jared D Wainwright[5], Kan Nakamoto[6], Perenlei Enkhbaatar[6], Kaitlyn A Cook[2], Kevin P. Francis[2], John Adams[2], Alexandra Stavrakis[2], Joseph C Wenke[7], Andrea M Kasko[1], and Nicholas M Bernthal[2]
[*]These authors contributed equally to this work

[1]Department of Bioengineering, University of California, Los Angeles, Los Angeles, CA 90095, United States
[2]Department of Orthopedic Surgery, David Geffen School of Medicine, University of California, Los Angeles, CA 90095, United States
[3]David Geffen School of Medicine at University of California, Los Angeles, Los California, CA 90095, United States
[4]University of California, Los Angeles, Los Angeles, Los California, CA 90095, United States
[5]Department of Orthopaedic Surgery and Rehabilitation, the University of Texas Medical Branch, Galveston, TX, 77555, United States
[6]Department of Anesthesiology, the University of Texas Medical Branch, Galveston, TX, 77555, United States
[7]Department of Orthopaedic Surgery and Rehabilitation, Shriners Children's Texas, the University of Texas Medical Branch, Galveston, TX, 77555, United States

Corresponding Author:
Christopher Hamad, MD
Department of Orthopaedic Surgery
University of California, Los Angeles
1225 15th Street – Suite 3144B
Santa Monica, CA 90404
Email: chamad@mednet.ucla.edu
Phone: 713-859-0308

Supplemental Materials and Methods

S1. Computational Modeling

To characterize hydrogel structure, the volumetric swelling ratio (Qv), polymer volume fraction (v2,s), and specific volume of polymer (v) were calculated. This allowed for the molecular weight between crosslinks (Mc) to be calculated. Using the respective values for Mc, the mesh size (x) was determined, from which the release kinetics and cumulative release can be modeled.

For the purposes of the model, mass swelling ration (qm) was set between 2 and 15. This was then used to calculate volumetric swelling ratio, Qv, using the bulk density (ρ_{BD}) for each PEG molecular weight (Table S1) and the known solvent density (ρ_S) of water.

$$Q_v = 1 + \left(\frac{\rho_{BD}}{\rho_s}\right) * (q_m - 1)$$

Equation S1

While the specific volume of the polymer, v, is ρ_{BD}/ρ_S, the volume fraction in the hydrogel swollen to equilibrium (v_2,s) is:

$$v_{2,s} = \frac{1}{Q_v}$$

Equation S2

This, along with the molecular weight (M_n) of PEG, is used to determine molecular weight of the polymer chains between two neighboring crosslinks, M_c:

$$M_c = \frac{1}{\left(\frac{2}{M_n}\right) - \left(\frac{\frac{v}{V_1} * \left(\log(1 - v_{2,s}) + v_{2,s} + X_1 * v_{2,s}{}^2\right)}{v_{2,s}{}^{1/3} - \frac{v_{2,s}}{2}}\right)}$$

Equation S3

The number of links in the chain, N, can be calculated knowing that the molecular weight of repeating units in the polymer chain (M_r) is 44.05 g/mol.

$$N = \frac{2 * M_c}{M_r}$$

Equation S4

The root mean square end-to-end distance of polymer chains between 2 neighboring crosslinks is then calculated, approximating the length of the bond along the polymer backbone, *l*, as 1.54 Å, and the polymer specific characteristic ratio, C_n, as 4.

$$(r_0{}^2)^{\frac{1}{2}} = l * (C_n * N)^{\frac{1}{2}}$$

Equation S5

Combining the information from Equations 2 and 5, we can calculate mesh size (ξ):

$$\xi = v_{2,s}{}^{-1/3} * (r_0{}^2)^{\frac{1}{2}}$$

Equation S6

Therapeutic drug diffusion coefficients, D_0, were determined using the Stokes Einstein equation. The hydrodynamic radius, r_s, for each therapeutic agent was approximated using a

hydrodynamic radius calculator (Table S2), and Y is a scaling factor that can be approximated to 1. These values were used to estimate the diffusion of each drug from the hydrogel (D_g) independently:

$$D_g = D_0 * \left(1 - \left(\frac{r_s}{\xi}\right)\right) * e^{-Y\left(\frac{v_{2,s}}{1-v_{2,s}}\right)}$$

Equation S7

The computational model was based on thin-film release, from hydrogels with a height of 2 mm, *L*, and a diameter of 6 mm. The amount of therapeutic drug released at time (t) versus the total amount of therapeutic drug released at time infinity was calculated using Equation 1.

In MATLAB, Equation 1 was used to calculate and plot cumulative release from the hydrogels for each of the therapeutic agents, from PEG of molecular weight 575, 700, and 2000 g/mol (**Fig. S6**).The computational model's predictions were used to narrow down PEG molecular weight candidates and provide insight into release kinetics of each of the different therapeutic agents

While the computational model predicted the release of bupivacaine, *in vitro* research quickly transitioned to lidocaine instead due to its faster time to onset. Due to their similar molecular weights and structures, the cumulative release profile of bupivacaine is expected to provide an estimate of lidocaine's release kinetics.

S2: Stability studies

Stability studies were designed to evaluate macromer solubility, chemical stability, and hydrogel gelation after storage for 1 day, 1 week, and 3 months at elevated temperatures. PEGDA 3350, TEMED, and APS samples stored in capped Eppendorf tubes were placed on a pre-heated heat block at 46°C. At each time point, ^{1}H NMR spectra of PEGDA and TEMED were recorded to evaluate degradation. PEGDA was diluted to the standard 35.5 wt%, and the solubility was observed to ensure that it had not prematurely polymerized. Finally, the samples were used to fabricate hydrogels to verify the gelation time had not significantly changed.

The percent acrylation of PEGDA at day 0 was 75% and remained unchanged at 3 months, indicating it had not auto-polymerized (**Fig. S2**). While both PEGDA and APS remained stable throughout the duration of the period, TEMED rapidly evaporated from the Eppendorf tube and was unable to be characterized. This indicates the need for improved packaging or a less volatile amine-based initiator for the system. Using the PEGDA and APS after each incubation period, and freshly prepared TEMED, the reagents solubilized easily and hydrogels gelled normally, indicating high stability of this system.

S3: Drug Stock Solutions

Each therapeutic agent was used to create fresh aqueous stock solutions. The drugs were weighed and diluted using DI H_2O to the desired concentrations. For *in vitro* experiments, concentrations of 10.5 mg/mL for bupivacaine (Alfa Aesar – Ward Hill, MA, USA) or lidocaine HCl (MP Biomedicals – Santa Ana, CA, USA), 191.6 mg/mL for tranexamic acid (Acros Organics

– Geel, Belgium), 5.2 mg/mL for vancomycin (ThermoScientific Chemicals – Waltham, MA, USA), and 92.9 mg/mL for tobramycin (TCI – Tokyo, Japan) were used. For *in vivo* murine experiments, each hydrogel contained 4 mg vancomycin, 4.57 mg tobramycin, 5.56 mg tranexamic acid, and 1.38 mg lidocaine. The mass of each therapeutic agent added was kept constant, regardless of final hydrogel volume. *In vivo* Merino sheep experiments used concentrations of 0.5375 g vancomycin, 0.6390 g tobramycin, 0.779 g tranexamic acid, and 0.1925 g lidocaine in each 35 mL hydrogel.

S4. Calibration curves

In vitro calibration curves of each therapeutic agent in water within relevant concentration ranges were obtained, to relate UV-Vis absorbance to drug concentration (**Fig. S3**). During *in vitro* kinetic release experiments, these calibration curves were used to plot concentration as a function of time, producing drug release profiles.

Because tobramycin and tranexamic acid lack UV absorbing chromophores, they required additional reagents to make them detectable via UV-Vis spectroscopy. A final concentration of 0.2% $FeCl_3$ (99+%, Acros Organics – Geel, Belgium) was added to drug dilutions and absorbance was measured at 370 nm to produce calibration curves for tobramycin and vancomycin (**Figs. S3B** and **S3D**). When determining *in vitro* release, the supernatant was transferred from the wells into individual Eppendorf tubes at each time point, and stored at -20°C until the last time point had been collected. After thawing, 400 µL of each sample was transferred into a 48 well plate and 54.55 µL of a 1.67% solution of $FeCl_3$ was added. The absorbance was recorded within 1 h using a plate reader (BioTek Synergy H1 Microplate Reader, Agilent – Santa Clara, CA, USA) at 370 nm, though absorbance remained stable for at least 24 h.

S5. Chromatography and spectrometry techniques

The chromatographic system consisted of Jasco (Tokyo, Japan) PU-2087 dual plunger pump, a high pressure dynamic mixer (Jasco, MX-2080-32) and a UV-visible tunable absorbance detector (Jasco UV-2075). The chromatograms were recorded using ChromNAV software (Jasco, Japan) to quantify peak heights and entire data analysis and processing. The mobile phase used in this system was degassed via sonication for 30 minutes at room temperature[21-23].

ESI-TOF measurements were carried out on a Waters LCT-Premier XE Time of Flight Instrument controlled by MassLynx 4.1 software (Waters Corporation, Milford MA). The instrument was equipped with the Multi Mode Ionization source operated in the electrospray mode. A solution of Leucine Enkephalin (Sigma Chemical, L9133) was used in the Lock-Spray.

Supplemental Figures

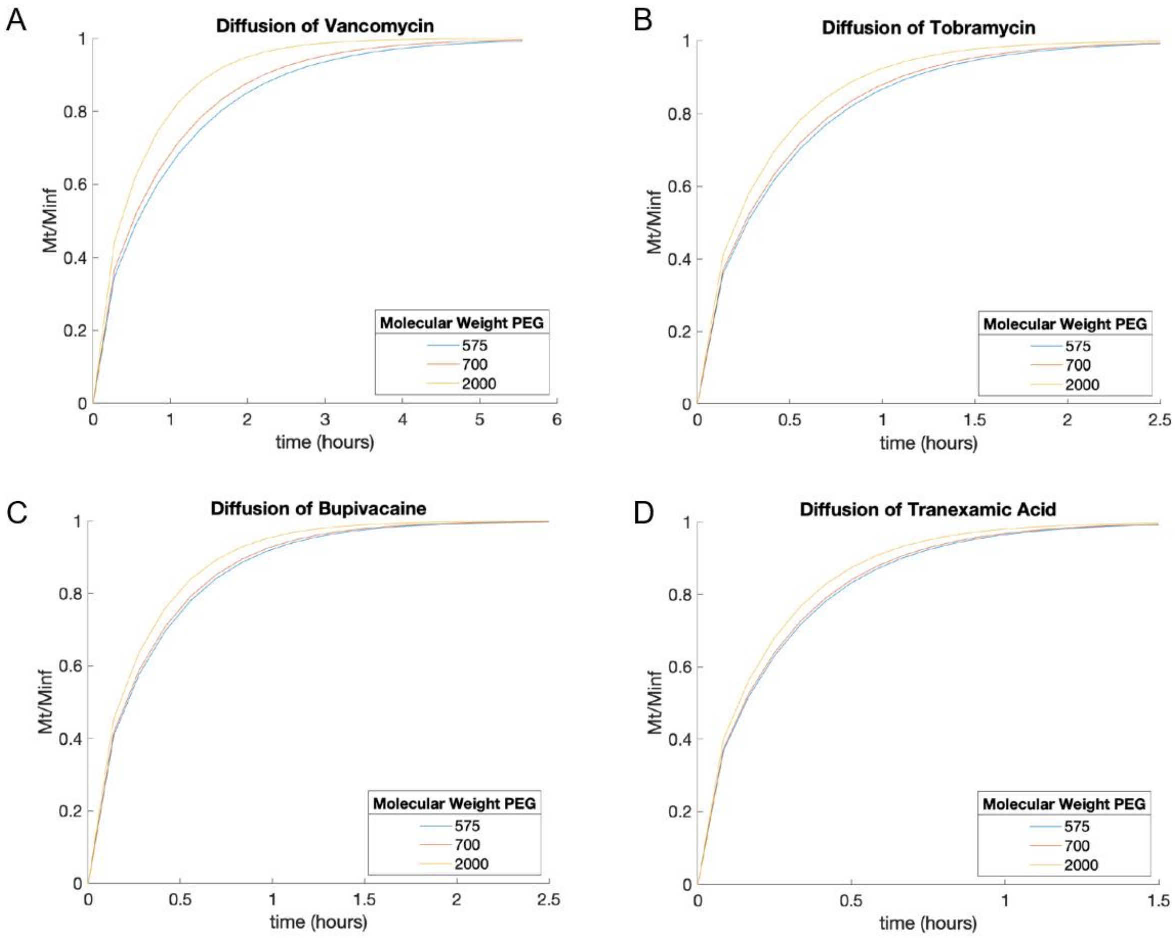

Supplementary Figure S1. Cumulative release computational models of (A) vancomycin, (B) tobramycin, (C) bupivacaine, and (D) tranexamic acid from varied molecular weight PEG hydrogels with 2 mm thickness.

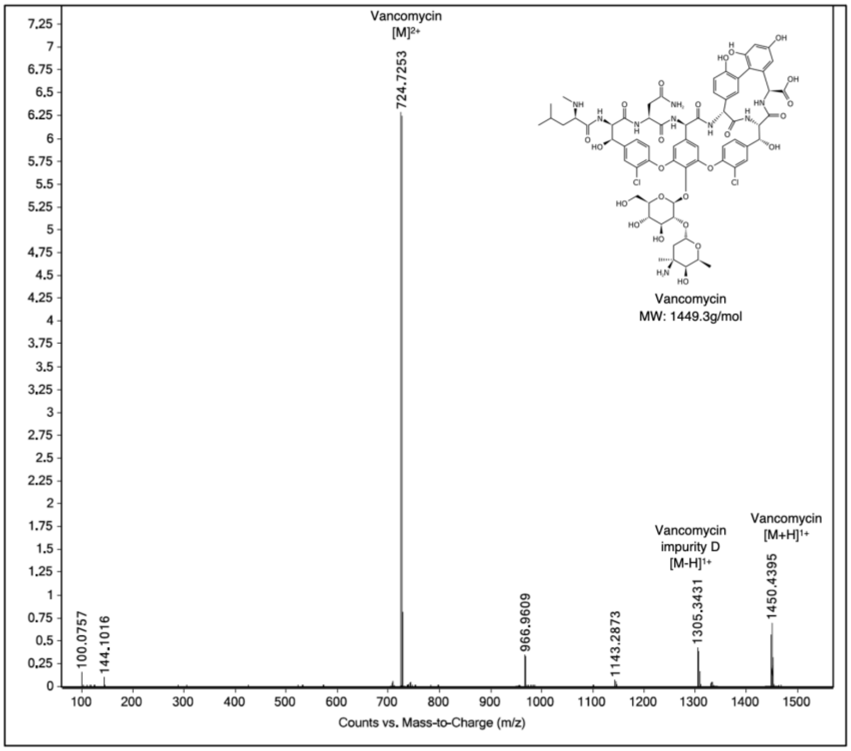

Supplementary Figure S2. Mass spectrum (electrospray ionization) of vancomycin, isolated from a hydrogel system containing all therapeutics. *Sample collected post-HPLC separation*.

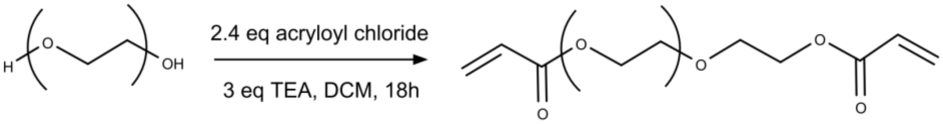

Supplementary Figure S3. Synthesis of polyethylene glycol-diacrylate (PEGDA).

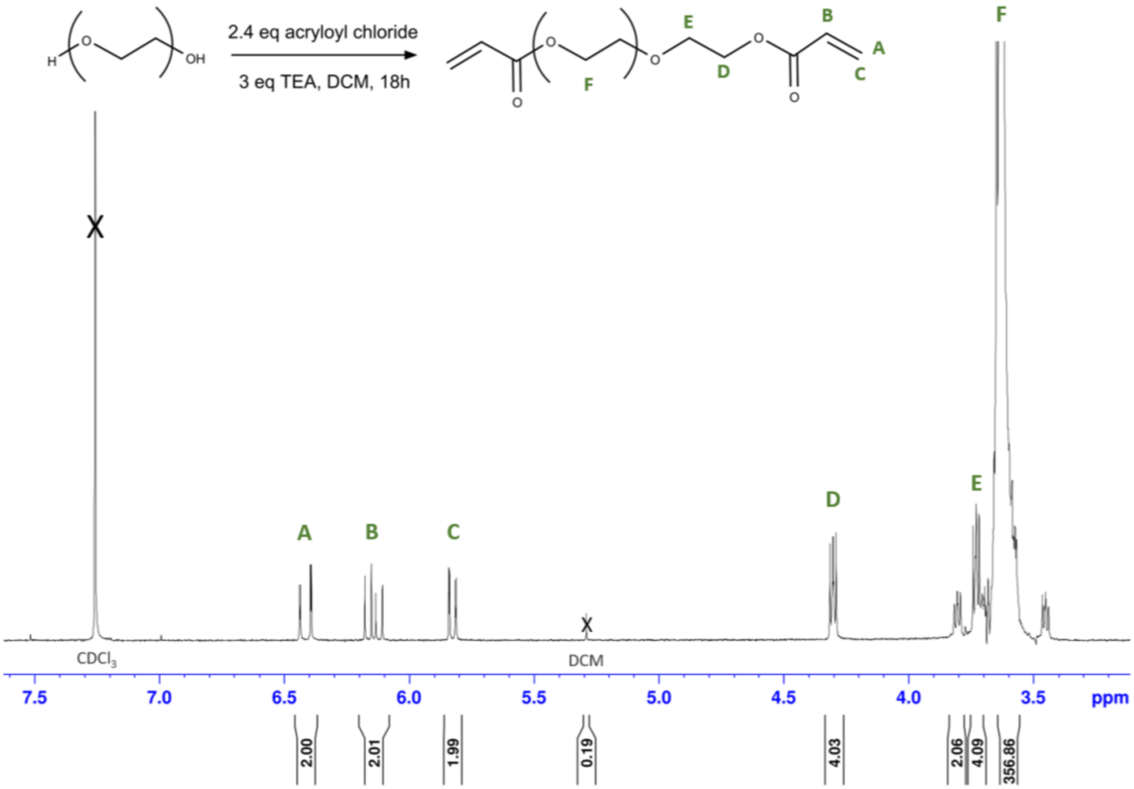

Supplementary Figure S4. ^{1}H NMR of PEGDA 3350 in $CDCl_3$.

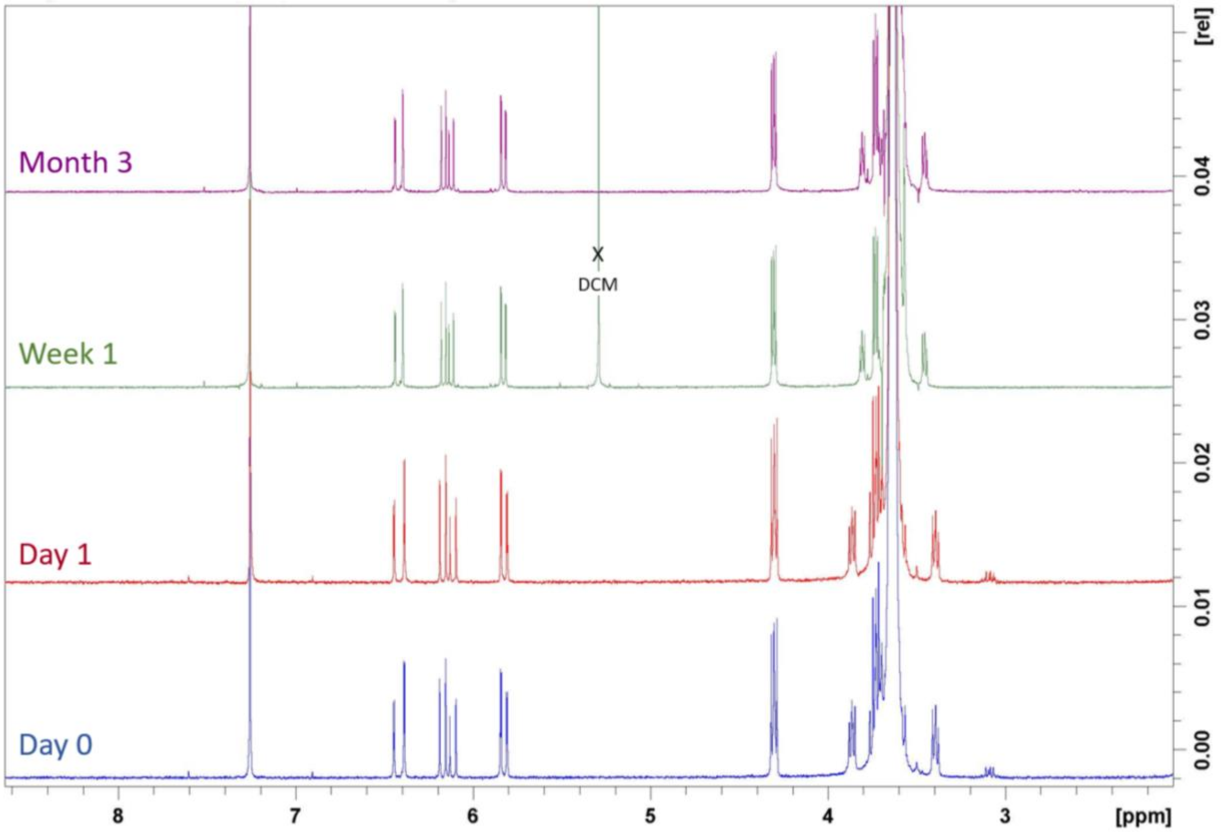

Supplementary Figure S5. ^{1}H NMR of PEGDA 3350 in $CDCl_3$ at 0 days (*blue*), 1 day (*red*), 1 week (*green*), and 3 months (*purple*).

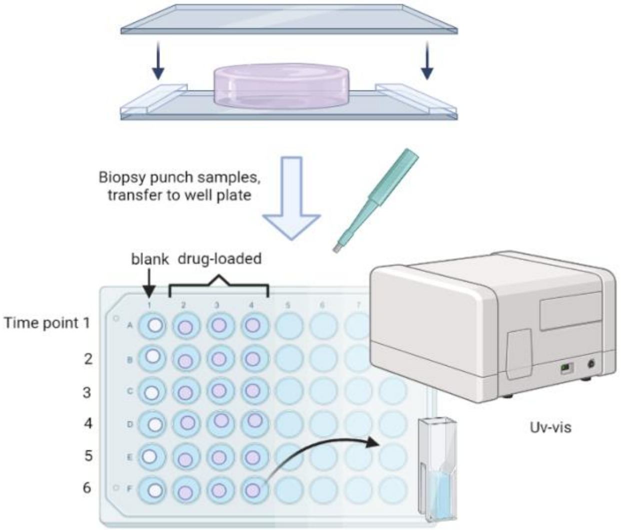

Supplementary Figure S6. *In vitro* therapeutic release characterization workflow.

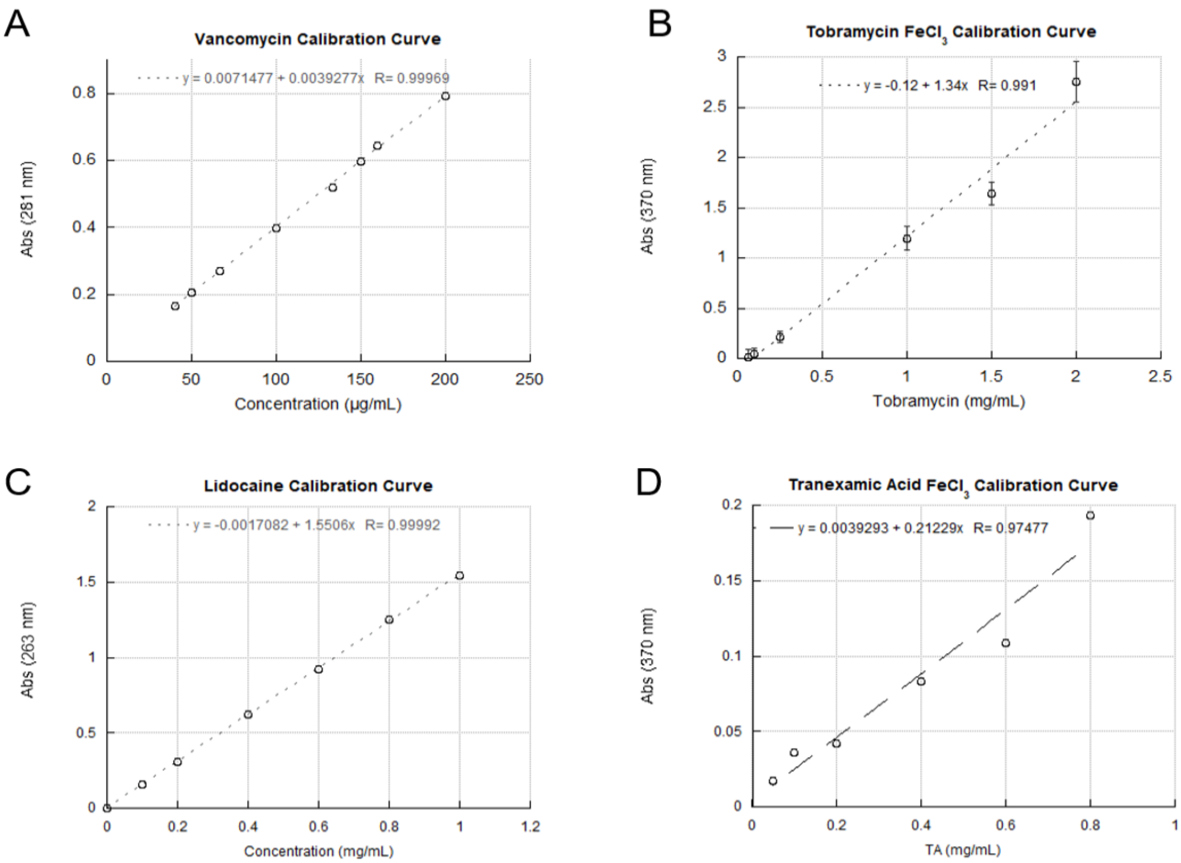

Supplementary Figure S7. Calibration curves relating concentration to absorbance of (**A**) vancomycin at 281 nm, (**B**) tobramycin with 0.2% $FeCl_3$ at 370 nm, (**C**) lidocaine at 283 nm, and (**D**) tranexamic acid with 0.2% $FeCl_3$ at 370 nm.

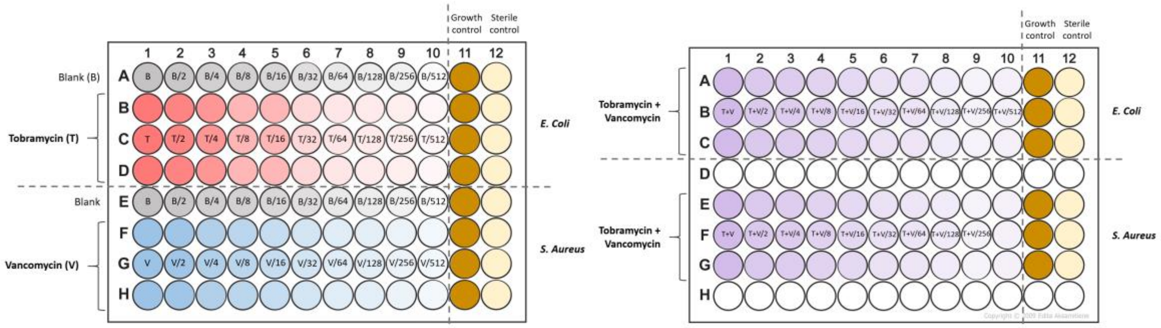

Supplementary Figure S8. Plate maps for LB broth microdilution assay to determine the minimum inhibitory concentration (MICs) of tobramycin-loaded hydrogels, vancomycin-loaded hydrogels, and dual-antibiotic-loaded hydrogels against *E. coli* and *S. aureus* strains. Positive growth controls (11) contain log-phase bacterial culture in media, and negative sterile controls (12) contain media alone.

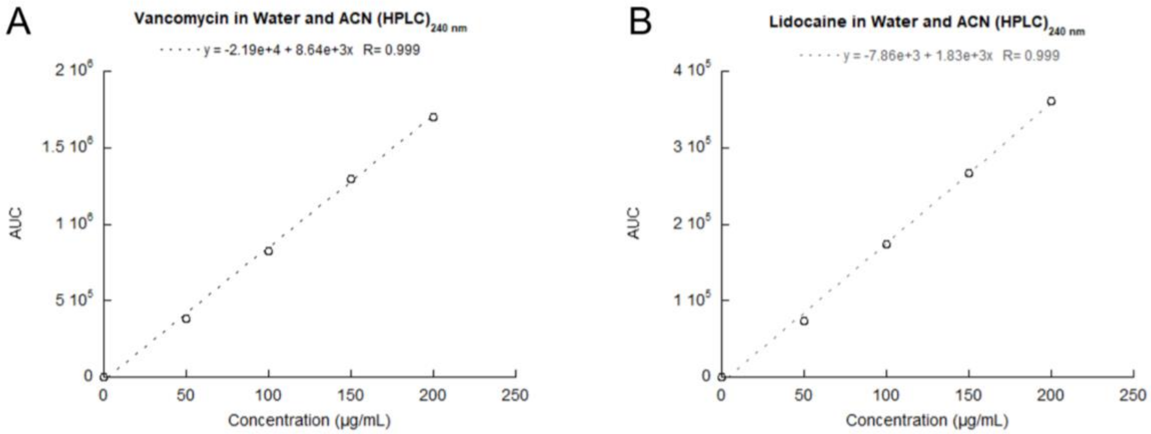

Supplementary Figure S9. HPLC calibration curves for area under the curve (AUC) as a function of (**A**) vancomycin and (**B**) lidocaine concentration, recorded with a mobile phase containing 70:30 (v/v) water and acetonitrile.

Glossary of Terms and Abbreviations

PEGDA: Polyethylene glycol diacrylate

PEG: Polyethylene glycol

APS: Ammonium persulfate

TEMED: Tetramethylethylenediamine

Qm: Mass swelling ratio

Qv: Volumetric swelling ratio

v2,s: Polymer volume fraction in the swollen hydrogel at equilibrium.

v: Specific volume of the polymer, calculated from the bulk density of PEG.

Mc: Molecular weight between crosslinks

Mn: Number-average molecular weight

Mr: Molecular weight of the repeating unit – 44.05 g/mol for PEG.

Cn: Characteristic ratio

l: Bond length between monomer units in PEG (1.54 Å).

ξ (xi): Mesh size

rs: Hydrodynamic radius

D0: Diffusion coefficient in water (free solution).

Dg: Diffusion coefficient in hydrogel.

TEG: Thromboelastography

LY30: Lysis at 30 minutes

MA: Maximum amplitude

G: Shear elastic modulus

tPA: Tissue plasminogen activator

IIa: Alpha-thrombin

HPLC: High performance liquid chromatography

UV-Vis: Ultraviolet-visible spectroscopy

ESI-MS: Electrospray ionization mass spectrometry

CFU: Colony forming unit

POD: Postoperative day

DI H_2O: Deionized water.
